# X-Rays in Cancer

**Published:** 1901-12-14

**Authors:** 


### X-RAYS IN CANCER.

At a recent meeting of the South-West London Medical Society, Mr. Cecil R. C. Lyster showed two cases of cancer which had been treated by exposure to the X-rays. The first was a case of epithelioma of the lower lip in a man aged 65. After five applications the ulcer appeared congested; there was a thin serous discharge, and a healthy granulating surface began to make its appearance. After an interval of about a week the treatment was resumed. Less congestion resulted this time, and the ulcer markedly diminished. After two more series of applications, that is five weeks from the commencement of the treatment, the ulcer had practically healed, although some slight thickening still remained towards the middle line. The second case was described in a note sent by Mr. Thomas Bryant. The patient was a lady, about 43 years of age, who had undergone an operation for the removal of a cancer of the breast in December, 1900. In August of this year she had appeared again with a recurrence in the site of the old operation, which had been observed about a month before, that is, about seven months after the operation. The clavicular flap was the seat of tubercles of a florid red colour, and the lower flap near the scar was dotted with like tubercles, while the upper flap near its axillary end was the seat of a raised tumour the size of a crown piece, the surface of which was breaking down, and there were other growths. There was much pain. After two weeks of treatment all pain ceased, and after the treatment had been carried on for three months, many of the active tubercles had entirely disappeared, and all were lessening, the other tumours were diminishing in prominence, and the skin which had been quite fixed was now movable. Of course, it does not do to speak hastily of cure, but at least it may be said that the acute form of the disease which existed at the commencement of the treatment had been entirely checked and exchanged for an atrophying condition. Another case of recurrent cancer now in hospital was also mentioned as undergoing the same treatment, in which similar changes appeared to be taking place.

### SALT A SUGGESTED CAUSE OF CANCER.

Dr. Braithwaite,[1] of Leeds, suggests that cancer results from the use of an excess of salt in the diet. It may be that several causes co-operate in the production of the disease; it may be that there is an over-nourished condition of the body resulting from eating too much, and especially too much meat; or a loading of the body with effete non-oxidised matters, as among those who lead indolent and indoor lives; or, again, that some local irritant or stimulant may determine the place at which cancer shall develop; but whatever may be the part played by these conditions, Dr. Braithwaite holds that the one factor which will be found to be operative in all cases is an excess of salt in the diet. In supporting his suggestion he points to the asserted rarity of cancer among the Jews, who do not eat pork; to the fact brought out by a recent research into the distribution of cancer in Buffalo, that the increase in the prevalence of cancer which has taken place there has fallen specially upon the foreign-born population, and particularly on the Germans, who eat much salted food; and to the generally asserted relation existing between prevalence of cancer and the consumption of large quantities of meat, which, he says, connotes large quantities of salt. At first sight what he says about the Jews might seem to incriminate the pig. But he points out that the pig is just the one domestic animal in which no case of cancer has yet been met with. Hence, if cancer occurs among those who feed on bacon, it is the salt and not the pig that does the mischief. Salt is a powerful stimulant to cell metabolism, as every watering-place physician knows. Such stimulation, however, according to Dr. Braithwaite, may be over-done. The farmer who over-manures his fields with artificial fertilisers finds weeds as well as crops grow up luxuriantly. So with the salt eater, in whose over-stimulated cells small provocations, which otherwise would have passed by without effect, set up infective overgrowths. It is an interesting speculation, which, however, seems to us to remain at present in the regions of pure hypothesis.

[1] Lancet, Dec. 7.

